# Infection following fractures of the proximal tibia – a systematic review of incidence and outcome

**DOI:** 10.1186/s12891-017-1847-z

**Published:** 2017-11-21

**Authors:** Ralf Henkelmann, Karl-Heinz Frosch, Richard Glaab, Helmut Lill, Christian Schoepp, Dominik Seybold, Christoph Josten, Pierre Hepp, Committee TRAUMA of the AGA-Society for Arthroscopy and Joint Surgery

**Affiliations:** 1 Department of Orthopedics, Trauma and Plastic Surgery, University of Leipzig, Liebigstr. 20, 04103 Leipzig, Germany; 2 Department of Trauma and Reconstructive Surgery with Divion of Knee and Shoulder Surgery, Sports Traumatology, Asklepios Clinic St. Georg, Lohmühlenstr. 5, 20099 Hamburg, Germany; 3 Departmet of Traumatology, Cantonal Hospital Aarau, Tellstrasse 25, CH-5001 Aarau, Switzerland; 4 Department of Trauma and Reconstructive Surgery, DIAKOVERE Friederikenstift gGmbH, Humboldtstr. 5, 30169 Hannover, Germany; 5 Departement of Orthopedic and Trauma Surgery, Berufsgenossenschaftliche Unfallklinik Duisburg, Großenbaumer Allee 250, 47249 Duisburg, Germany; 6 Department of General and Trauma Surgery, University Bergmannsheil Bochum, Ruhr-University Bochum, Bürkle-de-la-Camp-Platz 1, 44789 Bochum, Germany

**Keywords:** Surgical site infection, Outcome after infection, Proximal tibia fracture, Tibia plateau fracture, Outcome after infection

## Abstract

**Background:**

To systematically review all available studies of operatively treated proximal tibia fractures and to report the incidence of superficial or deep infection and subsequent outcomes.

**Methods:**

A systematic review of the literature in Medline, Cochrane, Embase and GoogleScholar was conducted to identify studies with cohorts of patients with infection after surgical treatment of proximal tibia fractures. Studies were included according to predefined inclusion and exclusion criteria. The studies were analysed for methodological deficiencies and quality of outcome reporting based on the Level of Evidence (LOE) and Coleman Methodology Scoring (CMS.)

**Results:**

In total 32 studies were included. There was heterogeneity between the studies, in terms of subject of the studies, outcome criteria, fracture type and classification, surgical techniques and length of follow-up. Therefore, no meta-analysis could be performed. The average CMS was 54.2 (range 36–75). The included studies were 25 case series (LOE IV), 6 were prospective cohort studies (LOE III) and one was a prospective randomized trial (LOE I). 203 (12.3%, range: 2.6–45.0%) infections occurred in the overall population (*n* = 2063). Those were divided into 129 deep infections and 74 superficial infections. Revision due to infection was reported in 29 studies, microbiological results in 6, respectively. 72 (55,8%) of 129 cases reporting outcome after deep infection had an unsatisfactory outcome with substantial limitations of the affected joint and leg.

**Conclusions:**

Postoperative infections are a challenge, sometimes requiring several revisions and often with a worse outcome. Further studies with structured study protocols should be performed for a better understanding of risk factors to improve treatment outcomes.

## Background

Proximal tibial fractures are common trauma injuries. Their severity is defined by fracture morphology and associated soft tissue injury. Management is challenging and patients are at risk for adverse outcomes [1].

In current literature, postoperative rates of surgical site infections (SSI) are between 3 and 45% [2–4]. This rate is high compared to SSIs with a rate of 2–3% of other fractures treated with open reduction and internal fixation (ORIF) [5–7] Why patients with proximal tibial fractures are prone to SSI compared to other fractures is unclear.

Furthermore, a change of the microbiological spectrum with regard to bacterial types and antibiotic resistance has been reported [8, 9]. Concomitant soft tissue injuries and open fractures complicate treatment through frequent operative revisions and higher infection rates.

Postoperative infections are a feared complication with an often unsatisfying outcome for the patient and possible loss of function in the affected region [10]. Patients with SSI have a higher mortality rate compared with patients without SSI and an extended hospital stay [11, 12]. To the authors’ knowledge, no previous review on this topic has been performed.

The purpose of this systematic review was to gain a more comprehensive understanding of the current infection rate and the outcome after infection of surgically treated proximal tibia fractures. Furthermore reoperation rates and if reported microbiology smears should be analysed.

## Methods

This systematic review followed the PRISMA (Preferred Reporting Items for Systematic Reviews and Meta- Analyses) guidelines for reporting systematic reviews and meta-analyses and the Cochrane Handbook for Systematic Reviews of Interventions. No review protocol was established prior to the begin of the search [13–15].

In February 2016 a systematic search in Medline (www.pubmed.com), Cochrane Library (www.cochranelibrary.com) and EMBASE using the following search terms and their combination with AND/OR: tibia* plateau fracture, proximal tibia fracture, tibia* head fractures, tibia head, knee, proximal tibia, infection, surgical site infection, surgical side infection, outcome, follow up and review was performed. The search included all available studies until the day of the search. Furthermore Reviews, editorials and opinion articles were used as potential sources of further references. The search strategy in Medline is pictured in Table 1.

**Table 1:** Pubmed.com search dated 2016.02.26

step	search terms	hits
#1	proximal tibia fracture	2082
#2	tibia* head fractures	552
#3	#1 AND infection	313
#4	#3 AND outcome	137
#5	#2 AND infection	58
#6	#2 AND outcome	84
#7	#1 “review”	216
#8	#2 “review”	57
#9	knee AND infection	9471
#10	knee AND surgical site infection	1603
#11	knee AND surgical side infection	138
#12	knee AND surgical site infection AND outcome	456
#13	#12 AND fracture	99
#14	proximal tibia	6655
#15	#14 AND infection	556
#16	#15 AND outcome	209
#17	#15 AND follow up	237
#18	tibia head	1131
#19	#18 AND infection	157
#20	tibia* plateau fracture	1355
#21	#20 AND infection	181
#22	#20 AND outcome	444
#23	#20 AND outcome AND infection	107

To include *‘grey literature’*, a search in Google Scholar with the search ‘tibial plateau fracture AND infection AND outcome’, ‘tibia head fracture AND infection AND outcome’ and ‘proximal tibia fracture AND infection AND outcome’ was performed.

Studies were included if they met the following criteria: (1) English or German language, (2) patients with tibial plateau fracture or tibial plateau fractures separable in the body of the text or in tables of any classification; (3) reported rate of SSI and outcome (4) studies with LOE of I through IV. Studies were excluded if they met one of the following criteria: (1) inclusion criteria were not met; (2) patients with tibia shaft fractures; (3) basic science only; (4) animal model only; (5) editorial, opinion, case report with less than ten patients, review or commentary.

Two authors (RH and PH) independently screened all retrieved items by tittle and abstract, than full text as necessary using the pre-determined selection criteria. Disagreements were resolved through discussion with CJ.

Data on study characteristics and design, level of evidence (LOE), demographic parameters, classification, surgical technique, infections, microbiology, revision surgeries, clinical follow-up and treatment outcomes were extracted by a single author (RH) from studies in a spreadsheet.

Statistical analysis was performed using the Statistical Package for Social Sciences (SPSS) v. 20 for Windows or RevMan v 5.3 (Nordic Cochrane Centre, Copenhagen, Denmark). All values are expressed as mean ± standard error of the mean (SEM) or range from minimum to maximum. If possible data will be pooled, an analysis of heterogeneity will be performed and a meta-analysis will be done. Furthermore this will be pictured in forest plots if possible.

### Coleman methodology scoring (CMS)

In addition to evaluating the studies for variables of interest, we also analyzed these studies for methodological deficiencies and quality of outcome reporting based on the recommendations given by Coleman et al. The score has ten sections with a maximum of 100 points [16].

### Outcome measures

The primary outcomes evaluated in this review were the rate of infection and the functional outcome after infection. The infections were graded into deep (DI) and superficial infections (SI) as classified in the articles. Secondary outcomes were results of microbiology smears and rate of reoperations.

## Results

### Included studies

**Fig. 1:**
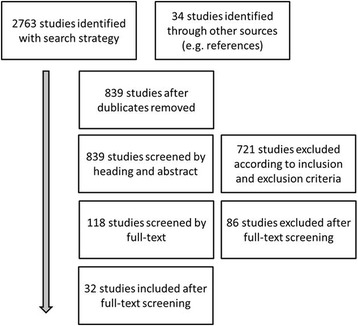
Flow-chart of included studies

A total of 839 titles and abstracts of articles were screened (Fig. 1). According to our inclusion and exclusion criteria and after removal of duplicates, 32 articles (2063 patients, Table 2) were included for this review. The study of Heppert et al. was excluded for this analysis due to the fact that their main inclusion criteria was a postoperative infection and their data would bias our results.

**Table 2:** Included studies in alphabetic order according to inclusion and exclusion criteria

author	PMID / DOI	study design	level of evidence	n	classification	open fractures included y/n	open fractures	%	infection y/n	number infection	%	deep infection y/n	number deep infection	% DI	Revision because of infection	Outcome
Babis [29]	21756337	retrospective case series	4	33	Schatzker	y	5	15.2	y	1	3.0	y	1	3	y	septic pseudarthrosis 1
Barei [30]	16882892	retrospective case series	4	83	AO	y	11	13.0	y	5	6.0	y	2	2,4	y	union 2
Barei [31]	15507817	retrospective case series	4	83	AO	y	11	13.3	y	15	18.1	y	7	8,4	y	union 7
Berber [32]	24377482	retrospective case series	4	16	Schatzker	y	2	12.5	y	2	12.5	n	0	0	n	good 2
Biggi [33]	20888560	retrospective case series	4	58	AO	y	3	5.2	y	4	6.9	y	1	1,7	y	good 1
Bucholz [34]	2537166	randomized controlled trial	1	40	Hohl	n	0	0	y	4	10.0	y	4	10	y	good 3 persistent infection 1
Chakraverty [35]	19516098	retrospective case series	4	16	AO	y	3	18.8	y	2	12.5	y	2	12,5	y	amputation 1 persistent infection 1
Cole [36]	15475848	retrospective case series	4	78	AO	y	22	28.2	y	2	2.6	y	2	2,6	y	union 2
Conserva [37]	26243524	retrospective case series	4	79	Schatzker	n	0	0	y	11	13.9	y	6	7,6	y	arthroplasty 1 good 5
Dallo’Oca [38]	23086660	retrospective case series	4	100	Schatzker	n	0	0	y	4	4.0	y	2	2	y	arthroplasty 1 persistent infection 1
Egol [39]	16056075	retrospective case series	4	53	Schatzker	y	16	30.2	y	3	5.7	y	3	5,7	y	nonunion 1 union 2
Engelbrecht [40]	4648423	retrospective case series	4	194	not mentioned	n	0	0	y	6	3.1	y	6	3,1	y	ankylosis 2 persistent fistula 1 union 3
Hadiukenych [41]	17332111	retrospective case series	4	54	AO	y	12	21.0	y	3	5.6	y	2	3,7	y	amputation 1 nonunion 1
Hutson [42]	9553864	prospective cohort	3	70	AO	y	33	47.1	y	5	7.1	y	3	4,3	y	persistent oedema 1 amputation 1 persistent infection 1
Jansen [43]	23661179	retrospective case series	4	22	AO	y	6	26.1	y	4	17.0	y	2	9,1	y	pseudarthrosis 2
Khatri [44]	10.1155/2014/589538	retrospective case series	4	65	Schatzker	n	0	0	y	6	9.2	y	3	4,6	y	delayed union 2 nonunion 1
Lin [19]	23754632	retrospective case series	4	251	AO	y	30	11.7	y	20	7.8	y	16	6,4	y	nonunion 3 malalignement 1 union 12
Marsh [4]	7744891	prospective cohort	3	20	Schatzker	y	7	35.0	y	9	45.0	y	2	10	y	ankylosis 1 union 1
Neogi [45]	26015609	prospective cohort study	3	61	AO	y	10	16.4	y	7	11.5	y	1	1,6	y	Stiffness 1
Ozkaya [46]	26021666	retrospective case series	4	22	AO	n	0	0	y	4	18.2	y	1	4,5	y	stiffness 1
Phisitkul [3]	17304060	retrospective case series	4	37	AO	y	6	16.2	y	8	22.0	y	8	21,6	y	amputation 1 satisfactory 7
Pun [47]	24600061	prospective cohort study	3	21	Schatzker	y	4	19.0	y	2	9.5	n	0	0	n	good 2
Rademakers [48]	17211262	retrospective case series	4	202	AO	n	0	0	y	11	5.4	y	11	5,4	y	arthrodesis 2 union 9
Sales [49]	24498816	retrospective case series	4	28	Schatzker	y	9	32.1	y	7	25.0	n	0	0	n	good 7
Stannard [50]	19753230	retrospective case series	4	52	AO	y	52	100	y	6	11.5	y	3	5,8	y	amputation 1 nonunion 2
Stannard [51]	14563009	prospective cohort	3	32	Schatzker	y	17	53.1	y	2	6.3	y	2	6,3	y	nonunion 1 union 1
Su [52]	15123954	retrospective case series	4	38	Schatzker	y	1	2.6	y	3	7.7	y	1	2,6	y	union 1
Wagner [53]	3749907	retrospective case series	4	32	AO	n	0	0	y	6	18.8	y	6	18,8	y	ankolysis 1 arthrodesis 5
Weaver [54]	22169068	retrospective case series	4	140	AO	y	20	14.3	y	15	10.7	y	15	10,8	y	nonunion 7 malalignement 8
Young [55]	8196973	prospective cohort study	3	45	AO	y	9	20	y	13	28.9	y	13	28,9	y	amputation 2 arthrodesis 3 ankylosis 8
Zhai [56]	24346508	retrospective case series	4	26	AO	y	8	30.8	y	4	15.4	y	1	3,8	y	good 1
Zhang [57]	22385447	retrospective case series	4	79	AO	y	7	8.9	y	9	11.4	y	3	3,8	y	bone graft and muscle flap 3

Of all included studies, 25 were case series (LOE IV), 6 were prospective cohort studies (LOE III) and one was a prospective randomized trial (LOE I). The average CMS was 54.2 (range 36–75) points.

Fractures were classified in descending order according to AO (59.4%), Schatzker (34.4%) and not mentioned/other in the article (6.3%).

### Open fractures

In total, if specified in the articles (*n* = 24, 1329 patients), 22.9% open fractures (*n* = 304) and 77.1% closed fractures (1024) were within the study population in those studies.

### Infections

Two hundred three (9.8%, range: 2.6–45.0%) infections occurred in the overall population (*n* = 2063). Those were divided into 129 deep infections and 74 superficial infections.

Studies without open fractures (*n* = 8, 734 patients) had an infection rate of 10.3% (3.1–18.8; 39 DI, 13 SI). Studies which included open fractures (*n* = 24, 1329 patients) resulted in an infection rate of 12.9% (2.6–45.0; 90 DI, 61 SI).

### Reoperation due to infection

In 29 studies, a reoperation due to infection was reported. Those studies had an average infection rate of 11.9% (2.6–45.0) and in total 192 infections within their study populations (129 DI, 63 SI). In those studies an average reoperation rate until the end of their follow-up between 2.1 and 5 reoperations per patient was reported.

### Microbiology

In six studies positive results of microbiological smears were reported. The most common bacterium was *Staphylococcus aureus* with or without resistance (methicillin-resistant *Staphylococcus aureus*, MRSA), followed by Enterobacter or Enterococcus species (Tables 2 and 3).

**Table 3:** Results of microbiological smears were given in six studies

	Engelbrecht [40]	Lin [19]	Marsh [4]	Cole [36]	Phisitkul [3]	Barei [31]
*Staphylococcus aureus*	x	x	x		x	x
MRSA		x		x	x	x
*Staphylococcus epidermidis*		x			x	
Streptococcus		x				
Pseudomonas aeruginosa		x				
Enterococcus		x			x	x
Enterobacteriaceae		x			x	x
Haemophilus influenzae					x	
no growth (if reported)		x		x	x	

### Studies with outcome information

Information about the outcome after infection was given in all included studies (Table 2) with 2063 patients (18.6% open fractures, range 0–100%). 203 infections (12.3% (2.6–45.0) occurred in this population including 129 (63.5%) deep infections. All patients with superficial wound infections (*n* = 74), were treated with wound care, oral antibiotics or single debridement if necessary. Furthermore three studies of those had no DI in their study population. All reported SI were reported with a good outcome withouth further specification.

In summary, 72 (55,8%) of 129 cases reporting outcome after deep infection had an unsatisfactory outcome with substantial limitations of the affected joint and leg. The most common limitation was non-union or pseduarthrosis (15.3%). Followed by joint stiffness due to operation (arthrodesis 7.8%), ankylosis (9.3%) or not specified sitffness (1.6%). The worst case an amputation was reported in 5.4% (Table 4).

**Table 4:** Outcome of 129 patients with DI

	Number	Percent
amputation	7	5.4
ankylosis	12	9.3
stiffness	2	1.6
persistent oedema	1	0.8
arthrodesis	10	7.8
malalignement	9	7.0
non-union / pseudarthrosis	20	15.3
persistent infection / septic pseudarthrosis	5	3.9
persistent fistula	1	0.8
arthroplasty	2	1.6
muscle flap	3	2.3
union / good / satisfactory	57	44.2
total patients	129	100

## Discussion

This systematic review of the literature on infections after surgical treatment of proximal tibia fractures included 32 studies and a total of 2063 patients. To our knowledge, this is the first review dealing with this topic.

Only one study investigated the outcome of infection after proximal tibia fractures [10]. Eighteen years ago, Heppert et al. included 52 patients with a mean age of 51.5 (18–89) years and a follow up between 11 and 13 months. Due to the infection 263 reoperation procedures (mean of 5.1 reoperations per patient) were performed. The individual outcome was an axial deformity (*n* = 15), ankylosis (*n* = 2), arthrodesis (*n* = 10) and amputation (*n* = 9). Hence they had a poor outcome in 69.2% (*n* = 36). In the present review we could extract 129 patients from 32 studies with further information concerning treatment outcome after postoperative infection. 72 patients (55.9%) had severe limitations and thus a poor outcome (Table 2).

Many studies are available concerning proximal tibia fractures. Literature reports a wide range of infection rates between 2.6–45%. The present review summarizes an infection rate of 12.3%. It is well known that infection rates are high for those fractures compared to an infection rate of 2–3% of other fractures treated with open reduction and internal fixation (ORIF) [5–7].

The most common bacterium was Staph. aureus with or without resistance (MRSA), followed by Pseudomonas, Staph. epidermidis, Enterobacteriaceae or Enterococcus species. The bacterial spectrum corresponded with the incidence in the literature [8]. In the face of a changing bacterial spectrum and increasing resistances a change of the current prophylactic antibiotic regimen could potentially close gaps. In particular the change of the bacterial spectrum in proximal tibia fractures was decribed by Morris et al [17].

Operative time and open fractures are independent predictors of postoperative infections [18, 19]. In the current study we could confirm a higher infection rate in studies which included open fractures. Nevertheless, little information regarding management of postoperative infections in tibial plateau fractures and their treatment outcome has been reported.

Fractures of the tibial plateau are usually severe injuries and include a wide variety of fracture patterns. The choice of approach is dictated principally by the fracture pattern, with consideration of the soft tissue envelope, patient factors, and associated injuries [20]. Due to the trauma mechanism, high energy trauma in young and direct impact in elderly patients, the soft tissue is usually traumatized twice, by the accident and sometimes by the subsequent operation. Accordingly, the rate of complications after fracture stabilization is high [21, 22]. In particular, the combination of fracture and soft tissue damage is challenging. Even without infection the functional outcome may be poor [23]. Limited range of motion and progressive osteoarthritis are possible complications which occur in 26.4% according to a recent study [24]. Infections even worsen the situation. Postoperative deep infections of the tibial plateau ended in most of the cases with a considerable functional loss.

### Limitations and future perspectives

One major limitation of the current review is the obvious heterogeneity between the studies, in terms of subject of the studies, outcome criteria, fracture type and classification, surgical techniques and length of follow-up. Moreover this was evident by the weak CMS of the included studies. The basic limitation of pooling data is the fact that a surgical site infection is a recorded side effect and not a subject of the included studies. Therefore, a meta-analysis with weighting of the studies or a determination of odds ratio of possible risk factors could not be performed. A pooling of reported percentage SSI rates in the studies could be done according to a narrative analysis.

If an infection occurred, inconsistent information with regard to time span until operation, number of reoperations, results of microbiological smears, treatment strategy and clinical outcome described by ROM or validated score could be retrieved. Furthermore it was seldom possible to comprehend if the infection occurred in an open or closed fracture. Also the degree of soft-tissue damage was seldom documented.

The limitations of the review provide guidance that could be used for future studies. Outcome was reported very inconsistently with different scores or range of motion. A few studies used an instrument to measure quality of life like SF-36 (36-item Short-Form General Health Survey) or KOOS. We would recommend giving detailed information about each patient with regard to comorbidities, long-term medication, time-span until operation, surgery duration, blood loss, and postoperative treatment protocol. Furthermore, risk factors for postoperative infections were characterized as describing reduced fitness, patient frailty and surgery complexity [25, 26]. We would also recommend a score which measures quality of life and function in daily living like SF-36 or KOOS [27, 28]. The KOOS is a patient-reported outcome measurement instrument. It is widely used in clinical trials and its psychometric properties have been validated. The score consists of five separately scored and validated subscales: KOOS Pain, KOOS Symptoms, Function in daily living (KOOS ADL), Function in Sport and Recreation (KOOS Sport/Rec), and knee-related Quality of Life (KOOS QOL). Additionally, in cases of infection, the involved bacterium, number of reoperations and detailed treatment strategy should be stated. This approach could provide independent entry points which could be positively influenced to reduce SSIs. The pooled review data are too weak to state a precise treatment algorithm for future patients.

## Conclusion

This review proved that in literature over all included studies infection rates of tibial plateau fractures are 4 to 5 fold higher than other fractures which were treated with ORIF. Furthermore reported outcome of patients with DI had considerable limitations of their affected leg. This review with It pointed out that there is still a lack in the treatment of tibial plateau fractures to prevent such high rates of SSI.

## Abbreviations

AO: Arbeitsgemeinschaft für Osteosynthesefragen
CMS: Coleman Methodology Scoring
DI: Deep Infection
KOOS: Knee injury and Osteoarthritis Outcome Score
LOE: Level of Evidence
MRSA: methicillin-resistant *Staphylococcus aureus*
ORIF: Open Reduction and Internal Fixation
PRISMA: Preferred Reporting Items for Systematic Reviews and Meta-Analyses
ROM: Range of Motion
SF-36: 36-item Short-Form General Health Survey
SI: Superficial Infection
SPSS: Statistical Package for Social Sciences
SSI: Surgical Site Infection
